# Public Health in Ireland

**Published:** 1920-07-03

**Authors:** 


### PUBLIC HEALTH IN IRELAND.

A great many people just now are prescribing nostrums for Irish distemper, varying from machine guns to divorce decrees. Unanimity in diagnosis or treatment is perennially absent. In this chaos it is refreshing to find a large body of experts, thoroughly representative of the community, in complete unison as to the establishment in Ireland of a Health Department. For a considerable time the Irish Public Health Council, consisting of seventeen members, under the chairmanship of Dr. Coey Bigger, has been formulating proposals to form the basis of legislation, and its report is now complete.

### Ireland Lags Behind.

In social progress Ireland has usually a way of finding herself following afar off, and dimly hoping that some day she may arrive. When England dispossessed Mother Gamp of her heritage, Ireland of her own choice stood aloof, with the inevitable result that the Irish Gamp trade was constantly being reinforced by refugees from Great Britain. Again, such medical benefits as accompanied National Health Insurance in England never crossed the Irish Channel. Voluntary county nursing schemes are almost unknown in Ireland. But several hundreds of public bodies, such as County and Rural Councils, Boards of Guardians, Pensions Committees, and National Health Insurance Societies, have a health function tacked on to their other multifarious duties. One result of this tinkering and overlapping is that it passes the wit of ordinary men to locate responsibility. The duties are seldom mandatory, so the usual attitude is one of masterly inactivity, and the patient muddles along as best he can, or, perchance, dies.

Some time ago a dispensary doctor was presented with a "red ticket," a document requesting him to call on a farm labourer lying sick in a remote part of his district. Some days elapsed before he found it convenient to answer the call. Indeed he arranged his visit to synchronise with a meeting of the hounds which he hoped to attend, and which would bring him that way. When ultimately he turned up, finding some difficulty in locating the patient, he inquired of a young farmer the way to proceed. The farmer was accommodating, and took charge of the steed while the doctor crossed a field or two, and finally a stile, which was to bring him to his man. Lo and behold, he found himself in a little country graveyard! The melancholy part of the story is that it is true. But it is more than a true story: it is a parable. It illustrates the Irish public health situation. There are Acts of Parliament and administrators without number, but incompetence, neglect, and national decadence are the resultant.

The Irish Public Health Council proposes to provide a remedy. There is recommended one National Health Department, to be administered by a Board, as in Scotland, and Public Health Councils, consisting of voluntary workers and paid officials, in every county and county borough. Their duties will be exclusive, and their powers extensive. The Chief Secretary is to be the Health Minister, and a permanent Chairman of the proposed Board will guide the policy of the Department. Local Health Boards will include in their purview the treatment of the sick poor, of insured persons, of tubercular or mental patients, of expectant and nursing mothers, of school children, and Infirmary Committees, Tuberculosis Committees, Insurance Committees, Asylums Committees will be reorganised or abolished.

### The Medical and Nursing Services.

The Council recommends drastic and far-reaching reform in respect of the medical and nursing services of Ireland, which admittedly are most unsatisfactory, alike to the members and to the public. The details of the proposed changes are, however, left over until a Health Ministry is actually established, so that local health authorities might co-operate in ensuring efficient service.

### Voluntary Hospitals.

Particularly interesting are the changes proposed with respect to voluntary hospitals. These, I venture to suggest, might be seriously considered as possibly applicable to the whole kingdom. It is proposed to utilise the voluntary hospitals of the country for the treatment of the sick, and to pay to them a capitation grant, partly from rates and partly from State grants. During the war wounded soldiers were sent to a large number of voluntary hospitals, which were most anxious to receive them on account of the income derived from the War Office for their treatment. In its estimates the Irish Health Council has allocated £150,000 for this purpose. If this scheme were adopted no hospital would be required to receive free patients, and the voluntary system would still be operative. In an interesting interview which I had with the Chairman I discovered his keen desire to utilise to the fullest possible extent the services of trained nurses. It is quite safe to prophesy that if legislation follows, health visitors will in Ireland be recruited solely from this source. Dr. Bigger regards three years' training and discipline in a public hospital as the only way to ensure an effectual health nursing service. By widening the nurse's outlook and providing her with a career, and incidentally with a competence and a pension, a high standard for probationers can be maintained.

The present system which prevails in England of throwing difficulties in the way of a nurse preparing to become a health visitor, and making University schools the exclusive place of training, is a direct incentive to the abandonment of nursing as a career by young women of refinement and reasonable ambition. IERNE.

